# Supplementary material for: Comparing Endovascular Treatment Methods in Acute Ischemic Stroke Due to Tandem Occlusion Focusing on Clinical Aspects
Source: Life (Basel). 2021 May 20;11(5):458. doi: 10.3390/life11050458 (PMC8160775; doi:10.3390/life11050458)
Supplement: Supplementary file 1 [file life-11-00458-s001.zip › life-1214794-supplementary.pdf]

Table S1. Evaluated parameters in patients with and without sICH.

	sICH (N = 7)	non-sICH (N = 94)	*p* value
Age, years, mean (±SD)	70 ± 10	67 ± 10	0.512
Gender, female, % (n)	28.6 (2)	39.4 (37)	0.572
Smoking, % (n)	57.1 (4)	61.9 (39)	0.806
Alcohol, % (n)	42.9 (3)	34.9 (22)	0.678
Hypertension, % (n)	42.9 (3)	72.8 (67)	0.093
Diabetes mellitus, % (n)	14.3 (1)	20.9 (19)	0.677
Atrial fibrillation, % (n)	0 (0)	21.3 (20)	0.173
Dyslipidaemia, % (n)	57.1 (4)	44.0 (40)	0.499
Previous stroke % (n)	0 (0)	19.3 (17)	0.234
API at admission, % (n)	14.3 (1)	23.0 (20)	0.595
OAC at admission, % (n)	0 (0)	9.2 (8)	0.402
Primary transport, % (n)	85.7 (6)	45.7 (43)	**0.041**
NIHSS baseline, median (IQR)	10 (5–17)	12 (9–16)	0.252
ASPECT score, median (IQR)	8 (7–9)	9 (8–9)	0.225
mCTA score, median (IQR)	3 (3–3)	4 (3–4)	0.089
IVT prior MT, % (n)	28.6 (2)	26.6 (25)	0.909
First pass effect, % (n)	33.3 (2)	54.9 (50)	0.304
TICI ≥ 2b, % (n)	71.4 (5)	84.0 (79)	0.390
Symptom onset to arterial puncture time median (IQR)	423 (334–785)	330 (228–623)	0.471
Puncture to revascularization time, median (IQR)	54 (48–63)	45 (32–65)	0.752
Symptom onset to revascularization, median (IQR)	518 (463–818)	393 (275–703)	0.444
Complications, % (n)	28.6 (2)	6.4 (6)	**0.036**
Distal emboli, % (n)	0 (0)	34.8 (32)	0.058
90 day mRS ≤ 2, % (n)	16.7 (1)	56.0 (51)	0.061
90 day mortality, % (n)	83.3 (5)	16.5 (15)	**<0.001**

Abbreviations: sICH, symptomatic intracranial hemorrhage; SD, standard deviation; API, antiplatelet inhibitor; OAC, oral anticoagulant; IQR, interquartile range; NIHSS, National Institutes of Health Stroke Scale; ASPECT, Alberta Stroke Program Early CT Score; mCTA, multiphase CT-angiography; IVT, intravenous thrombolysis; TICI, Thrombolysis in Cerebral Infarction; mRS, modified Rankin Scale.
